# Supplementary material for: Reconciling Mining with the Conservation of Cave Biodiversity: A Quantitative Baseline to Help Establish Conservation Priorities
Source: PLoS One. 2016 Dec 20;11(12):e0168348. doi: 10.1371/journal.pone.0168348 (PMC5173368; doi:10.1371/journal.pone.0168348)
Supplement: S1 Dataset — (ZIP) [file pone.0168348.s002.zip › Taxa/Serra Norte/SN_2013/N4E/Lista N4E-95.pdf]

CAVIDADE N4E-0095

Classe	Ordem	Fam/Outros	Gên/Outros	Espécie	S-U	U-S	Zon.
Annelida	Hirudinea			sp.1		2	P
Annelida	Oligochaeta			jovem	5		P
Arachnida	Acari	(Oribatida)		sp.1		X	E
Arachnida	Acari	(Oribatida)		sp.3		X	E
Arachnida	Acari	(Parasitengonina)		sp.2		X	E
Arachnida	Acari	Anystidae	*Erythracarus*	sp.1		X	E
Arachnida	Acari	Ixodidae	*Amblyomma*	sp.1		X	E
Arachnida	Acari	Laelapidae		jovem	X		E/P
Arachnida	Acari	Podocinidae	*Podocinum*	sp.1	X		E
Arachnida	Acari	Prostigmata		sp.49	X		P
Arachnida	Acari	Trombiculidae		sp.41	X		E/P
Arachnida	Amblypygi	Charinidae	*Charinus*	sp.1	3	3	P
Arachnida	Amblypygi	Phrynidae	*Heterophrynus*	*longicornis*	1	2	E/P
Arachnida	Araneae	Corinnidae	*Tupirinna*	sp.1	1		E
Arachnida	Araneae	Ctenidae		jovem		1	E
Arachnida	Araneae	Filistatidae		jovem		X	E
Arachnida	Araneae	Pholcidae	*Mesabolivar*	*aurantiacus*		X	E
Arachnida	Araneae	Pholcidae	*Mesabolivar*	sp.1	X	X	E
Arachnida	Araneae	Scytodidae	*Scytodes*	*eleonorae*		1	E
Arachnida	Araneae	Segestriidae		jovem		X	E
Arachnida	Araneae	Theridiosomatidae	*Plato*	sp.1	X	X	E/P
Arachnida	Opiliones	Escadabiidae		sp.2	X	X	P
Arachnida	Opiliones	Sclerosomatidae	*Prionostema*	sp.1	2		
Arachnida	Palpigradi	Eukoeneniidae	*Allokoenenia*	sp.1	X		P
Arachnida	Pseudoscorpiones	Olpiidae		sp.1		X	E
Chilopoda	Scolopendromorpha	Scolopocryptopidae	*Tidops*	sp.1	1		P
Chilopoda	Scolopendromorpha	Scolopocryptopidae		sp.2	1		P
Chilopoda	Scutigeromorpha	Pselliodidae		jovem		3	E/P
Diplopoda	Polydesmida	Chelodesmidae		sp.3	1		P
Diplopoda	Polydesmida	Fuhrmannodesmidae		sp.1	X	X	E
Diplopoda	Polydesmida	Pyrgodesmidae		sp.1	X	X	E/P
Diplopoda	Spirostreptida	Pseudonannolenidae	*Pseudonannolene*	jovem	6		P
Entognatha	Collembola	Cyphoderidae		sp.1	X		P
Entognatha	Collembola	Entomobryidae		sp.3		X	E
Entognatha	Collembola	Isotomidae		sp.1	X		E/P
Entognatha	Collembola	Paronellidae		sp.1	X		E/P
Entognatha	Collembola	Paronellidae		sp.4		X	E
Entognatha	Collembola	Sminthuroidea		sp.2	X		P
Entognatha	Diplura	Campodeidae		sp.1		X	E
Insecta	Blattodea	Polyphagidae		jovem		2	E
Insecta	Coleoptera	Carabidae		sp.3	X	X	E/P
Insecta	Coleoptera	Chrysomelidae	Bruchinae	sp.1	X		P
Insecta	Coleoptera	Chrysomelidae		sp.4	X		E
Insecta	Coleoptera	Staphylinidae		sp.48		X	E
Insecta	Coleoptera	Staphylinidae		sp.51	X		P
Insecta	Diptera	Cecidomyiidae	Cecidomyiinae	sp.		X	E
Insecta	Diptera	Ceratopogonidae		sp.	X	X	P
Insecta	Diptera	Chironomidae		sp.	X	X	E/P
Insecta	Diptera	Culicidae	*Culex*	sp.	X	X	E/P

Insecta	Diptera	Dolichopodidae		sp.	X		E
Insecta	Diptera	Drosophilidae	*Drosophila*	*eleonorae*		X	E
Insecta	Diptera	Phoridae	Metopininae	sp.	X		E
Insecta	Diptera	Phoridae	Phorinae	sp.	X		E
Insecta	Diptera	Psychodidae	Phlebotominae	sp.	X	X	E/P
Insecta	Diptera	Psychodidae	*Telmatoscopus*	sp.	X		P
Insecta	Diptera	Sciaridae		sp.	X	X	E
Insecta	Heteroptera	Cydnidae		sp.		X	E
Insecta	Heteroptera	Notonectidae		sp.	X		
Insecta	Heteroptera	Reduviidae	Emesinae	sp.		3	E
Insecta	Heteroptera	Reduviidae	Triatominae	sp.	1	16	E/P
Insecta	Heteroptera	Veliidae	*Paravelia*	sp.	X	X	E/P
Insecta	Homoptera	Cixiidae		jovem	X		E
Insecta	Hymenoptera	Chalcidoidea		sp.1		X	E
Insecta	Hymenoptera	Formicidae	*Acromyrmex*	sp.1	X		E/P
Insecta	Hymenoptera	Formicidae	*Camponotus*	sp.1	X	X	E/P
Insecta	Hymenoptera	Formicidae	*Crematogaster*	sp.1	X		E
Insecta	Hymenoptera	Formicidae	*Gnamptogenys*	sp.2	X		P
Insecta	Hymenoptera	Formicidae	*Gnamptogenys*	sp.3		X	E
Insecta	Hymenoptera	Formicidae	*Hypoponera*	sp.1	X		P
Insecta	Hymenoptera	Formicidae	*Paratrechina*	sp.2		X	E
Insecta	Hymenoptera	Formicidae	*Pheidole*	sp.2	X		E
Insecta	Hymenoptera	Pompilidae		sp.	X		E
Insecta	Hymenoptera	Vespidae		sp.2		X	E
Insecta	Isoptera	Termitidae	*Nasutitermes*	sp.	X	X	E/P
Insecta	Lepidoptera	Galacticidae		sp.		X	E
Insecta	Lepidoptera	Tineoidea		sp.1		X	E
Insecta	Neuroptera	Myrmeleontidae		jovem		X	E
Insecta	Orthoptera	Gryllidae		sp.	1		E
Insecta	Orthoptera	Phalangopsidae	*Paraclodes*	jovem	10	15	E
Insecta	Orthoptera	Phalangopsidae	*Phalangopsis*	sp.	37	72	P
Insecta	Psocoptera	Amphipsocidae	*Calocaecilius*	sp.1		X	E
Insecta	Psocoptera	Epipsocidae	*Epipsocus*	sp.2	X		P
Insecta	Psocoptera	Psyllipsocidae	*Psyllipsocus*	sp.1		X	E
Insecta	Psocoptera	Ptiloneuridae	*Triplocania*	sp.9		X	E
Insecta	Thysanura	Nicoletiidae		sp.1	X	X	P
Gastropoda	Pulmonata	Bulimulidae	*Naesiotus*	sp.	X	X	E/P
Gastropoda	Pulmonata	Systrophiidae	*Happia*	sp.	X	X	E/P
Malacostraca	Decapoda			sp.		1	P
Malacostraca	Isopoda	Dubioniscidae		sp.1		X	E
Malacostraca	Isopoda	Philosciidae		sp.1	X		E
Symphyla		Scutigerellidae		sp.	X		P
Amphibia	Anura	Leptodactylidae	*Leptodactylus*	cf. *vastus*	2		E/P
Amphibia	Anura	Pipidae	*Pipa*	sp.	8	4	P
Amphibia	Anura	Strabomantidae	*Pristimantis*	cf. *fenestratus*	1		E/P
Amphibia	Anura			sp.		100	P
Mammalia	Chiroptera	Emballonuridae	*Peropteryx*	*kappleri*	2	1	E/P
